# MAIT cells in liver inflammation and fibrosis

**DOI:** 10.1007/s00281-022-00949-1

**Published:** 2022-05-31

**Authors:** Hema Mehta, Martin Joseph Lett, Paul Klenerman, Magdalena Filipowicz Sinnreich

**Affiliations:** 1 Peter Medawar Building for Pathogen Research, Nuffield Department of Medicine, University of Oxford, South Parks Rd, Oxford, OX1 3SY UK; 2 Liver Immunology, Department of Biomedicine, University Hospital Basel and University of Basel, Basel, Switzerland; 3 Translational Gastroenterology Unit, John Radcliffe Hospital, Oxford, OX3 9DU UK; 4 Department of Gastroenterology and Hepatology, Basel University Medical Clinic, Cantonal Hospital Baselland, Liestal, Switzerland

**Keywords:** MAIT cell, Hepatocyte, Liver inflammation, Liver fibrosis, Cytokine, MR1

## Abstract

Mucosal-associated invariant T cells or MAIT cells are an abundant cell type in humans and especially so in the liver. MAIT cells are a subset of T lymphocytes that sit at a bridge between innate and adaptive immunity, so-called innate-like or “unconventional” T cells. The specificity of their antigen receptor (T cell receptor or TCR) is for the conserved major histocompatibility complex (MHC)-related molecule MR1, which presents a modified bacterial metabolite from the vitamin B2 biosynthesis pathway – this allows them to respond in the presence of many bacteria or yeast. MAIT cells also possess an array of cytokine receptors, which allows triggering independently of the TCR. The combination of such signals drives their functionality – this means they can respond to a range of stimuli and likely play a role not only in infection or inflammation, but also under homeostatic conditions.

In this review, we will look at the question of what MAIT cells are doing in the normal liver and how they behave in the setting of disease. These questions are of relevance because MAIT cells are such a distinctive cell type enriched in the liver under normal conditions, and their modulation could be of therapeutic benefit. The recent discovery that they appear to be involved in liver fibrosis is particularly of interest in this context.

## Introduction

The immunology of the liver is complex and also still poorly understood. It is complex as, like other barrier sites, the liver is highly exposed to microbial products and also external antigens from food – these need to invoke an appropriate response such as tolerance or anergy. Failure to do so would lead to very high levels of immune activation. The tolerogenic function of the liver has been long recognized since the ground-breaking work of Roy Calne over 50 years ago, showing that livers can be transplanted across conventional MHC barriers [1, 2]. More than this, once transplanted the liver can impact on the acceptance of other organs. Hence the concept that the liver is a “tolerogenic” organ was established.

On the other hand, the presence of microbes in such proximity, not just bacteria but also viral threats, means the liver must be poised to allow a rapid response to a mucosal breach or invasion. Hence the normal rules of immunology must apply, i.e., innate responses should be mounted rapidly and help to trigger and guide subsequent adaptive immunity. The role of the innate response is highlighted in the description of the liver as a “firewall” by the group of Andrew McPherson [3]. Failure to control invasive infection from the gut at this firewall can lead to disastrous systemic infection.

These issues are not unique to the liver – similar balanced responses in the presence of a microbiome are managed in the gastrointestinal tract, the skin, and the lung. However, the composition of immune mediators and their function in the liver is unique. Among these features are the specific types of lymphocytes seen. This review is focusing on mucosal-associated invariant T (MAIT) cells, but other lymphocyte subtypes are also enriched in the liver, including T cells such as gamma delta (γδ) T cells and other innate-like cells such as iNKT (invariant Natural Killer T) cells. What these groups of cells have in common is a balance between innate and adaptive features – we hypothesize that this balance allows a regulation to be established within the cell to account for rapid triggering as needed, but also a homeostatic response in the absence of threat. This within-cell balance may be very important in the liver given the enormous volume of the organ and the scattering of the cells within the lobules [4–7]. Therefore, each cell must make its own decision depending on local inflammatory cues as to how to behave, rather than relying on complex regulatory circuits to maintain immune tolerance.

## What is a MAIT cell and how is it recognized?

Although MAIT cells were discovered around 30 years ago, it took a while for their abundance to be fully recognized, and they are still often either ignored or not segregated from other T cell populations, despite their distinct features. This is gradually changing with widespread coverage of their biology and also the use of single-cell RNAseq approaches pointing to their distinctive transcriptional features.

**Fig. 1:**
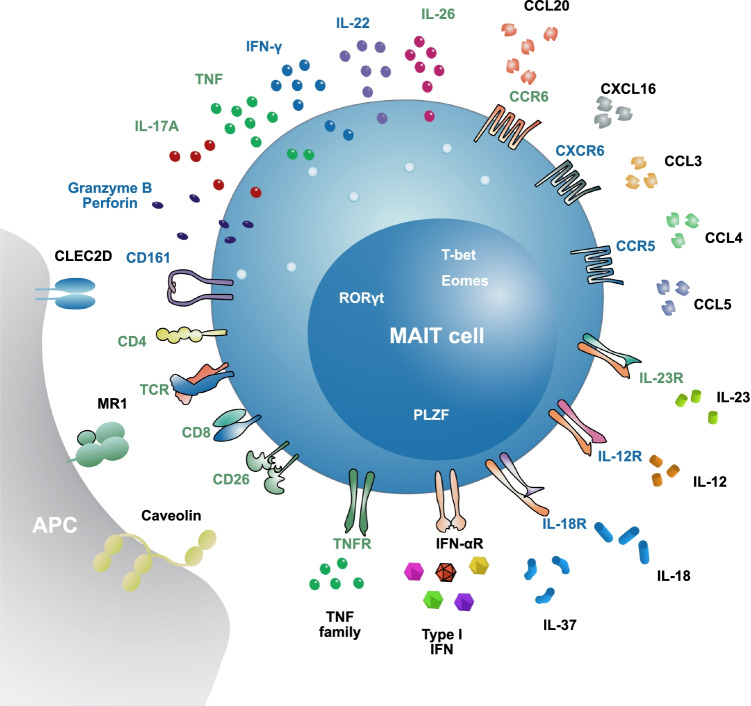
Surface receptors and secreted proteins characterizing MAIT cells. Names of proteins expressed in NK and T cells are in blue; of proteins expressed mostly on T cells in green; and of proteins expressed by a variety of cells in black. An antigen-presenting cell (APC) is represented in gray. Transcription factors are depicted in the MAIT cell nucleus. Known ligands for the receptors expressed on MAIT cells are also shown

The initial description focused on the abundance of a specific T cell receptor in early TCR sequencing studies [8, 9]. Subsequent studies by Olivier Lantz demonstrated the enrichment of such cells at mucosal sites in humans and also mice and gave them their name [10]. The TCR associated with a MAIT cell is the Vα7.2 alpha chain, combined with a restricted repertoire of J alpha chains and a limited number of beta chains [9, 11]. The TCR alpha chain antibody is therefore a key part of the staining panel typically used to identify MAIT cells in humans by flow cytometry (FACS) [12]. However, it is not sufficient as many other conventional T cells will express this chain. A second marker that is useful is C-type lectin CD161 (expressed from gene *KLRB1*). CD161 is strongly expressed on MAIT cells, and indeed almost all CD8^+^ T cells that express the highest levels of CD161 (typically written as CD161^++^ or CD161-bright) are MAIT cells [13, 14]. Around 90% of MAIT cells in humans express CD8, and 10% are double negative for co-receptors CD8 and CD4, with usually a small number expressing CD4 (this may vary) [12, 13]. There are other features of the cell that can be used to identify them that correlate typically with high levels of CD161. These include staining for CD26 (dipeptidyl peptidase IV/DPP4) [15], interleukin (IL)-18 receptor-α [13], or the intracellular transcription factor PLZF (*ZBTB16*) [16]. Such combinatorial staining patterns are valuable in FACS and immunohistochemistry (IHC)/immunofluorescence (IF) approaches. A summary of MAIT cell surface markers is shown in Fig. 1.

A complementary way to define MAIT cells is with the use of MR1-antigen tetramers. Once the key ligand 5-(2-oxopropylideneamino)-6-D-ribitylaminouracil (5-OP-RU) had been defined (see below), by the groups of Rossjohn and McCluskey [17, 18], they were able to create soluble, fluorescent molecules containing MR1 and 5-OP-RU [19]. Such molecules identify almost exactly the same population of T cells as described above, especially in blood where the staining of CD3^+^CD161^++^Vα7.2^+^ cells is very similar to that of CD3^+^CD161^++^tetramer^+^ cells. Thus, the definition of a MAIT cell as a Vα7.2^+^CD161^++^ cell has been modified somewhat to include tetramer binding cells. There are some exceptions to this rule, and there is some variability in the antigen targeted, but generally staining protocols using the TCR identify MR1-restricted microbial reactive cells. This is also true in tissue, although a great deal of care needs to be taken to avoid aberrant staining and autofluorescence. Of note, there are some exciting new T cell populations which bind MR1 presenting an as yet unidentified ligand from tumors – so-called MR1-T cells [20, 21]. These may represent an interesting new anti-tumor population, but they use a different set of TCRs and have different phenotypes, so they should not be confused.

## Triggering and known functions of MAIT cells

Following their description, further work on the MAIT cell TCR showed that the cells recognized a microbial product and that the restricting or presenting molecule was MR1 [10]. MR1 is MHC-like in structure and highly conserved in mammals, with extensive cross-species recognition [22, 23]. The groove of MR1 where a peptide would normally sit in a classical MHC molecule is much smaller, and a non-peptide ligand was eventually identified as the pyrimidine 5-OP-RU [18]. 5-OP-RU is a modified version of 5-amino-6-ribitylaminouracil (5-A-RU), an intermediate on the pathway to forming riboflavin (vitamin B2) in microbes – most gram-negative and gram-positive bacteria and many yeast – a pathway not present in their mammalian hosts. 5-OP-RU is relatively labile and is created by coupling of 5-A-RU with methylglyoxal, a molecule that is constantly produced in mammalian cells from lipid peroxidation and glycolysis, the latter being the principal source [24].

Recognition of the MR1-ligand complex on its own is sufficient to trigger some MAIT functions, but optimal triggering requires co-stimulation with cytokines, such as IL-12, IL-18, or interferon-alpha (IFN-α). Thus, engagement with antigen-presenting cells (APCs) that have taken up microbes such as *Escherichia coli*, which produce the ligand, leads to strong activation, including secretion of cytokines, cytotoxicity, and proliferation [25]. Triggering in the absence of cytokines, however, produces an interesting partial response with more limited cytokine secretion; studies in men and mice have revealed that this pattern of responses appears to favor tissue repair or wound healing [26–30]. Indeed mice which lack MR1 (and therefore do not generate MAIT cells in the thymus) show a deficit in wound healing in the skin, where the TCR triggering is driven by local commensal organisms [26].

MAIT cells are very sensitive to triggering by cytokines alone, independently of their TCR [31], allowing them to respond to viral infections, such as influenza and hepatitis C virus (HCV) [32], and likely other inflammatory settings such as steatohepatitis. The key cytokines involved are type I interferons, IL-12, IL-18, IL-15, and also members of the TNF superfamily [28]. Recently, TNF has been shown to be critical for such activation after triggering by an adenovirus vaccine [33].

The response pattern of MAIT cells is driven by their underlying transcriptional status. Of note, they express T-bet and provide a type 1 function as classically associated with effector CD8^+^ T cells. They also constitutively express RORγt, which drives a type 17 functionality [13, 14, 34]. Although this is visible in their phenotype and function, following stimulation of MAIT cells from human blood, the levels of IL-17A and F secreted ex vivo are quite low. IL-17 expression can be upregulated in tissues and under pathologic conditions [35–39]. Mice show a different pattern, where MAIT cells split into type 1 and type 17 subtypes [40], but in humans, no real functional subtypes have yet been identified, even if there are some subtle differences between CD8^+^, double-negative, and the rare CD4^+^ MAIT populations [41, 42].

Rationalizing all this, we have hypothesized a model where MAIT cells in barrier tissues, including the liver, are continuously exposed to a certain level of the soluble ligand. In the absence of any inflammatory cues – i.e., danger signals as proposed by Polly Matzinger in the 1990s [43] – they will perform a homeostatic role with a wound repair profile. In the presence of low levels of cytokines, e.g., IFNs, TNF, and IL-18, driven through pattern recognition receptors, they will rapidly upregulate effector functions including cytokine and chemokine release and cytotoxicity. This places them among the first responders in any inflammatory and infectious setting.

Data in support of this model have recently been obtained from our labs, where we assayed the ability of liver-derived cells to present MAIT cell ligands and the presence of such ligands in the peripheral and portal circulation [7]. These experiments demonstrated that human hepatocytes are potent presenters to MAIT cells and the ligand is clearly detectable in blood, as a consequence of production in the GI tract. The consequences for MAIT cell function in a physiologic state remain to be determined.

### Liver enrichment and liver tissue-homing properties of MAIT cells

**Fig. 2:**
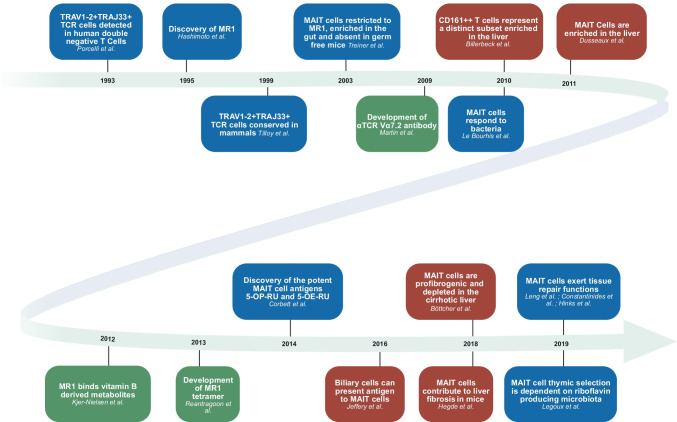
Timeline highlighting some of the most relevant findings in studies of MAIT cells in the liver. Findings in blue are crucial for the whole MAIT cell field; green boxes show crucial tool development; and studies in red concern the liver. See text for references

Enrichment of MAIT cells in the liver has been noted by many groups, using slightly different markers as the field has evolved as described above. Among T cells, MAIT cells are present in human blood at around 5–10% of CD8^+^ T cells on average, although the range is very wide [12, 13]. CD8^+^ T cell populations themselves are enriched in the normal liver over CD4^+^ T cells, and MAIT cells have been described as regularly up to 30–40% of CD8^+^ T cell populations [13, 35], so a marked enrichment. This is greater than seen for most other organs assayed, including the gut and lung. A timeline highlighting some most relevant findings in studies of MAIT cells in the liver is shown in Fig. 2.

The same redistribution of MAIT cells to the liver has been seen in the cow [44]. This is of interest because among a range of animals (pig, sheep, mouse, macaque); the cow shows a similar phenotype and frequency of MAIT cells to an adult human. Also, recent work demonstrated MAIT-like cells in the bat *Pteropus alecto* with characteristics and phenotype similar to human MAIT cells [45]. Mice in contrast have very low levels of MAIT cells (about 100-fold lower than humans) and in contrast much higher levels of iNKT cells (about 100-fold that of humans) [46]. In mice, iNKT cells are highly concentrated in the liver. Mouse iNKTs (and also human iNKTs) share many features of human MAIT cells, with strong sensitivity to cytokines and a marked type 1 cytokine/cytotoxicity profile, but lack the RORγt expression and type 17 biology [46]. Overall though, these cells likely compensate for each other and fill, with subsets of γδ cells, a similar ecological niche in tissues. Importantly though, the phenotypes seen in some mouse knockout lines show that even these relatively low levels of MAITs in mice play a critical and also a nonredundant role [47].

**Fig. 3:**
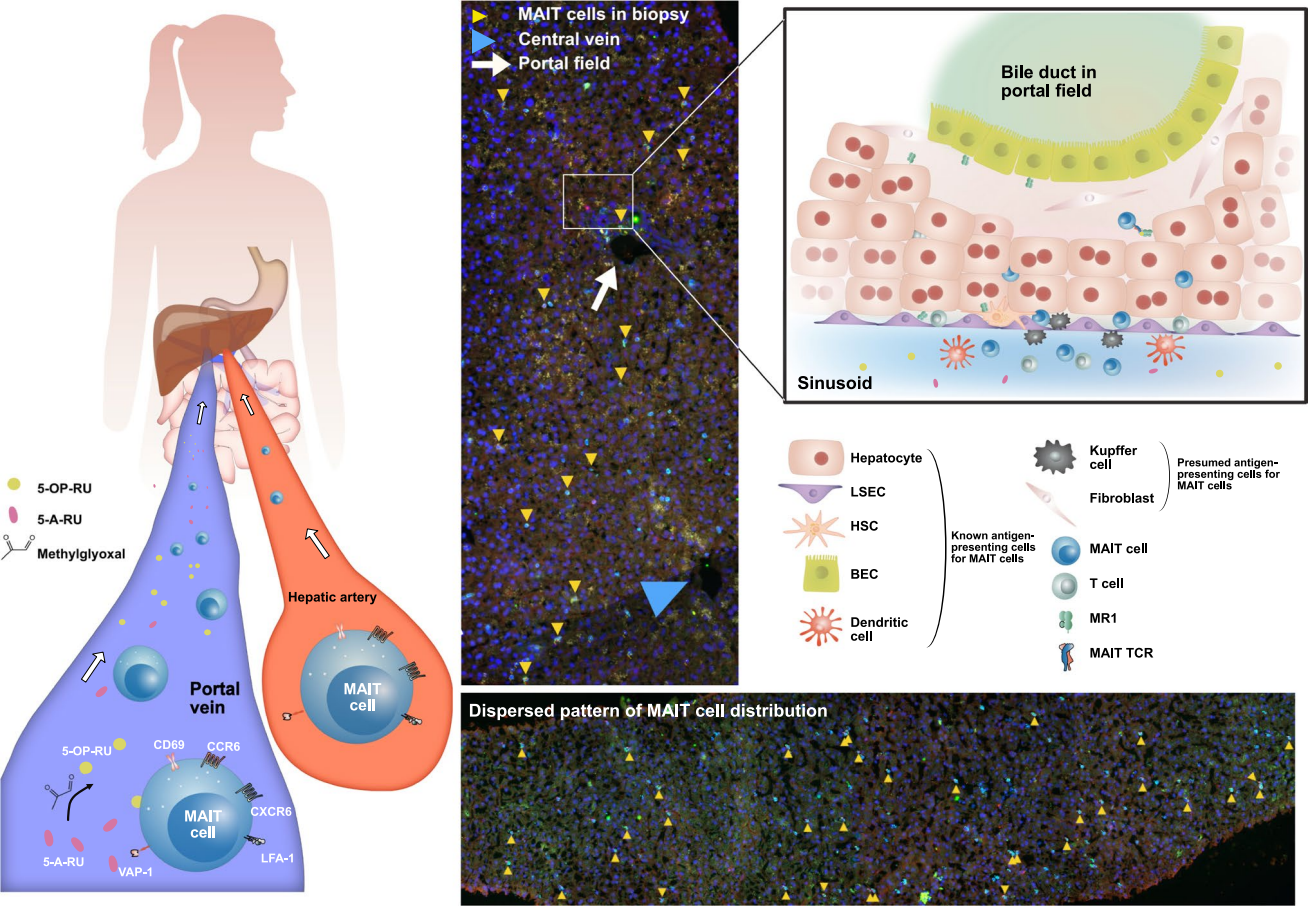
MAIT cell homing and distribution in healthy human liver tissue. Left panel: homing of MAIT cells to the liver via the portal vein and the hepatic artery. Examples of homing receptors and integrins (CD69, CXCR6, CCR6, LFA-1, VAP-1) are depicted on a MAIT cell. Gut bacteria-derived MAIT cell stimulatory metabolites are entering the liver via the portal vein. IF staining of tissue sections from liver biopsies without histopathological abnormalities: co-localization of CD3, TCR Vα7.2, and IL18-Rα identifies MAIT cells (yellow arrowheads; methods as in reference [7]). Right panel: cellular interactions of MAIT cells in the liver. Various liver cells including hepatocytes, biliary epithelial cells (BECs), hepatic stellate cells (HSCs), and liver sinusoidal endothelial cells (LSECs) activate MAIT cells in an MR1-dependent manner

The distribution of MAIT cells in the normal liver has not been consistently defined, but certainly includes widespread localization around the lobules, rather than a specific portal location [4–7]. Some inconsistencies in visualization are partly because of the staining issues described above and availability of antibodies that work effectively in fixed tissues, as well as a lack of truly normal tissues. In the most recent study, based on IF staining of healthy liver tissue for CD3, Vα7.2, and IL18Ra, 5% of MAIT cells were found in portal fields, with most of the remaining ones localized dispersedly in the sinusoidal environment as supported by digital analysis [7]. In comparison to other T cells and also NK cells, the dispersed distribution with moderate proneness towards portal fields represents a unique feature of MAIT cells, as non-MAIT T cells were more frequently (~ 30%) found within portal fields, and NK cells, another immune cell population present in the liver, were dispersed like MAIT cells, but without proneness towards portal fields. Figure 3 shows examples of liver biopsy specimens derived from healthy human liver tissue indicating the dispersed pattern of MAIT cell distribution; it also shows homing pathways and cellular interactions of MAIT cells, as described in [7] and discussed in this review. If a lobular distribution is correct, this would fit well with the mouse model of iNKT cells [48]. These have been found to patrol sinusoids, using CXCR6, a receptor which is highly expressed also in MAIT cells. Studies of acute seronegative liver failure also suggested a parenchymal distribution, compared to chronic inflammatory disease [4], further arguing for a natural dispersed pattern which changes in disease. In most disease settings, there is a marked loss of MAIT cells in the liver (see below), so the change in pattern may represent not only redistribution but also decreased survival.

MAIT cells also express chemokine receptors such as CCR6 and CCR5 and so will localize to inflammatory cues [49, 50]. This may explain their redistribution in inflammatory disease, e.g., tuberculosis [51, 52]. MAIT cells migrating to infected tissues have also been suggested by an elegant study conducted with *Salmonella enterica* by the laboratory of Vincenzo Cerundolo, where the authors could show an oligoclonal proliferation of MAIT cells, a decrease in their frequency in circulation, followed by a recovery after treatment [53].

High-level expression of CCR6 on MAIT cells, which is very unusual for circulating CD8^+^ T cells, contributes to their tissue homing. CCR6 is the receptor for CCL20, produced in mucosal tissues and the liver. Hence, CCR6 is believed to home T cells to the mucosa and liver, two tissues highly enriched for MAIT cells [47, 54]. CCL20 is expressed by biliary cells, so some interaction there is expected, and within portal tracts there does appear to be an interesting association between MAIT cells and biliary epithelium [4]. Biliary cells have also been shown to present bacterial Ag to MAIT cells [4], consistent with a protective role in this sub-compartment. Recently, we have identified additional interactions of MAIT cells with liver cells [7]. Hepatocytes, hepatic stellate cells (HSCs), and liver endothelial cells (and also biliary cells) were found to activate MAIT cells in an MR1-dependent manner, with hepatocytes being the most efficient. These cells also promoted the formation of active 5-OP-RU Ag if exposed to 5-A-RU, indicating that a process of both Ag accumulation and its presentation by APCs can occur in the sinusoidal environment rich in the dispersedly localized MAIT cells (see also Fig. 3) [7].

Besides CCR6, half of the circulating MAIT cells express surface CXCR6 [50], which is uncommon in circulating memory CD8^+^ T cells. The ligand for CXCR6 is CXCL16, known to be expressed on liver sinusoidal endothelial cells [48, 55]. The CXCR6-CXCL16 axis is thus another homing signal directed to the liver where the expression of CXCR6 has been shown to favor CD8^+^ T cell survival [56]. Hence, the dual expression of CCR6 and CXCR6 is likely to be part of the mechanism explaining the very high abundance of MAIT cells in the human liver.

The question is often asked as to why MAIT cells are so enriched in the liver and ultimately this is central to their role in disease. The answer lies partly in their distinct transcriptome and phenotype, which is present in the thymus as part of their initial development. Recent findings demonstrate that gut bacteria-derived 5-OP-RU controls the intra-thymic development of MAIT cells in mice [57]. “Naïve” MAIT cells exit the thymus with a phenotype similar to that of effector memory or even tissue-resident memory cells. Overall, the natural expression of integrins such as LFA-1 and VAP-1, as well as chemokine receptors as described above, favors liver recruitment and retention (as indicated, this is not unique to the liver). MAIT cells in the liver have a phenotype and transcriptome profile which resembles that seen in blood MAIT cells [35], with the main difference being some features of local activation, e.g., high expression of CD69, known to promote T cell retention in peripheral tissues [58]. Thus, MAIT cells are generated (“preset”) with a highly adapted phenotype – and possess functions appropriate for local host defense and potentially for homeostasis.

## Potential role of MAIT cells in liver homeostasis and tissue repair

As described above, TCR triggering of MAIT cells, in the absence of cytokines, induces a tissue repair gene expression program. Strong indications for such MAIT cell function stem from an in vivo model of skin injury [26], in vitro cultures of colon cells [28], and analysis of MAIT cells isolated from the gut, lung, and blood [27–29, 59]. Interestingly, a recent report describes a tissue repair signature also in TCR stimulated liver-derived MAIT cells [30], opening the possibility that liver MAIT cells are involved in liver homeostatic and possibly regeneration processes. Their possible role in homeostasis is supported by the presence of MAIT cell stimulatory metabolites in the sera of healthy individuals [7]. These metabolites would likely lead to constant low-level MR-1 mediated activation, rationalizing the known activated phenotype of MAIT cells in the liver. Additional evidence for a protective role of MAIT cells in the liver stems from a mouse study in which absence of MAIT cells exacerbated liver disease [60]. Further studies in mouse models of liver regeneration are needed to address a possible involvement of MAIT cells. We will next address the role of MAIT cells in liver disease.

## MAIT cells in the inflamed liver

Liver inflammation can be caused by various triggering conditions, including viral infection, autoimmunity, excess fatty infiltration due to the metabolic syndrome, or exposure to alcohol, and by medication. Numerous immune cell types including both innate and adaptive immune cells are known to contribute to intrahepatic inflammation in various liver diseases as reviewed elsewhere [61–66]. We will focus on what is known about the contribution of MAIT cells to inflammatory processes in the liver and will provide a limited description of data on blood MAIT cells in the discussed patient populations. A very thorough review of the contribution of both peripheral and liver MAIT cells to the pathogenesis of chronic liver disease was recently published [67].

Liver MAIT cells secrete large amounts of pro-inflammatory and fibrogenic cytokines, including IFN-γ, TNF-α, and IL-17. This cytokine expression profile, combined with MAIT cell abundance in the liver and their potential of cytotoxicity, suggests a prominent role of MAIT cells both in liver inflammation and fibrogenesis. Secretion of IL-17 by MAIT cells follows repetitive IL-12 stimulation or occurs in response to IL-7, a cytokine secreted by hepatocytes under inflammatory conditions [4–6, 35, 68]. Other local factors, including gut-derived metabolites and also cytokines (e.g., those secreted by dendritic cells and monocytes within the liver) will likely additionally affect the function of intrahepatic MAIT cells.

The frequency of circulating MAIT cells is generally decreased in patients with liver diseases. As their phenotype was consistently described as hyperactivated and exhausted, it is assumed that the cells become apoptotic because of continuous stimulation [5, 6, 69–71]. Alternatively, it has been hypothesized that MAIT cells might be recruited to the inflamed liver, as has been the case of other inflamed organs [72–74], thereby decreasing in the periphery. However, as will be described in detail below, enrichment of MAIT cells in the inflamed liver has rarely been found, and their levels frequently appear to be decreased. Nevertheless, the observed decrease in the liver could at least partially be due to technical issues, as many studies assess MAIT cell frequency versu*s* CD3^+^ T cells by flow cytometry, and not their absolute amount in liver tissue, which might explain the discrepancy between studies. Thus, whether the same decrease in frequency as seen in the circulation holds also true for intrahepatic MAIT cells in patients with liver diseases is not entirely clear.

### Viral hepatitis

In patients with viral hepatitis, the peripheral and intrahepatic MAIT cells were generally reported as depleted; this was most robustly shown for circulating MAIT cells in HCV infection [32, 69, 75]. Whether the intrahepatic depletion represents a true loss of MAIT cells or just their dilution by increased infiltration of conventional T cells into the infected liver remains a matter of debate. As the frequency of MAIT cells was inversely correlated to the inflammatory grade seen in liver histology in hepatitis C [69], the second scenario is very likely. Blood MAIT cells are reduced in their function and also exhausted in HCV infection [69, 76], while liver MAIT cells were found more activated and had more cytotoxic potential than their peripheral counterparts [69]. In these patients, sustained IFN-γ production was shown for blood MAIT cells in response to cytokine – but not TCR-mediated stimulation. If a similar maintained IFN-γ production capacity also applies to liver MAIT cells, this would argue for their sustained antiviral and at the same time also proinflammatory/immune-pathological activity in the highly inflammatory milieu of the infected liver. Interestingly, highly specific antiviral therapy reconstituted intrahepatic MAIT cell frequencies and also decreased their hyperactivated and cytotoxic phenotype [69], a phenomenon not seen in the case of blood MAIT cells [77].

The data for patients with hepatitis B virus (HBV) infection are less consistent. An early study reported maintained MAIT cell levels in the blood of infected patients, with increased CD38 expression, reflecting activation, that decreased to baseline levels upon antiviral treatment. All 6 investigated patients had high levels of intrahepatic MAIT cells, although these patients were undergoing treatment and hence had suppressed viral loads [78]. Other studies similarly found increased expression of activation markers and functional exhaustion of blood MAIT cells in HBV patients, with PD-1 on MAIT cells positively correlating with plasma HBV-DNA levels, suggesting exhausted MAIT cells contribute to the failure of viral control [70, 79]. Percentages of both peripheral and intra-sinusoidal MAIT cells were however significantly reduced [79, 80]. Interestingly, Liu et al. reported recently that MAIT cells exhibit strong MR1-dependent cytotoxicity against HBV-transfected hepatocytes and that conjugated bilirubin promotes MAIT cell activation and apoptosis [80]. In patients coinfected with HBV and hepatitis delta virus (HDV), MAIT cells were found in an activated state, possibly due to increased IL-12 and IL-18 secretion by activated monocytes, resulting in functional impairment and progressive loss of peripheral and intrahepatic MAIT cells [81].

Taken together, viral hepatitis appears to lead to MAIT cell activation (likely cytokine-mediated) which may contribute to the observed immune pathology, while at the same time resulting in MAIT cell depletion, a process that was shown to be – at least in the liver of HCV patients—reversed by successful antiviral treatment. Although a clear in vitro antiviral activity against HBV and HCV has been shown for MAIT cells, their activated/exhausted phenotype might contribute to the failure of viral clearance and development of viral infection chronicity. Studies thoroughly assessing both the ex vivo phenotype and activation potential of liver-derived MAIT cells in acute as well as chronic viral hepatitis are lacking, thus leaving it open as to what extent these cells contribute to antiviral and inflammatory processes occurring specifically in the liver of the patients.

### Autoimmune liver diseases

Autoimmune liver diseases encompass autoimmune hepatitis (AIH), primary sclerosing cholangitis (PSC), and primary biliary cholangitis (PBC). AIH is characterized by lymphoplasmocytic infiltrates that affect portal tracts and also spill into the liver lobules (termed “interphase hepatitis”), while inflammation in PSC and PBC primarily affects bile ducts.

A study assessing the whole spectrum of autoimmune liver diseases found a lower frequency of MAIT cells in the liver and circulation. Blood MAIT cell frequency tended to decrease with increased fibrosis stage in the tissue and the remaining cells secreted TNF and IL-17A, a profibrogenic cytokine [6]. Liver Vα7.2^+^ cells, as assessed by IHC (no other markers were used to unequivocally identify MAIT cells), were decreased in numbers in PSC and PBC, but remained at healthy control levels in AIH livers. Blood MAIT cells showed signs of exhaustion (high expression of activation markers CD38 and HLA-DR, inhibitory molecules CTLA-4, TIM-3, and terminal exhaustion marker CD39), a phenotype that could be mimicked by repetitive stimulation by IL-12 and IL-18; additionally, repetitive IL-12 stimulation led to IL-17 secretion by MAIT cells [6], again arguing for mostly cytokine-mediated activation of MAIT cells in the disease context.

In patients with AIH, a marked decrease in circulating MAIT cells, increased cytotoxic function, and impaired ex vivo activation was described, especially in patients with advanced fibrosis, with these abnormalities persisting in patients in remission under treatment [82]. Liver MAIT cells were assessed by IHC for either Vα7.2 or CD161; cells positive for these markers increased in treatment-naïve AIH patients, both in liver lobules and portal tracts. However, since the quantitation was also here only done using one marker at a time, it remains unclear if these cells were bona fide MAIT (or rather infiltrating conventional Vα7.2^+^ T) cells. Also, the number of liver samples in the healthy control group was limited to two [82].

In patients with PSC, histologically characterized by periductal fibrosis that may be accompanied by infiltrates of inflammatory cells, MAIT cells were preserved within bile ducts (sampled by performing biliary brushing during endoscopic retrograde cholangiopancreatography), but strongly declined in the circulation. Peripheral MAIT cells were less responsive to *E. coli* stimulation, but still responded to cytokines [83]. In IHC performed on explanted end-stage PSC livers, Vα7.2^+^ cells were present in similar amounts as in healthy control liver tissues, whereas CD3^+^ cells were strongly increased, leading to lower relative amounts of Vα7.2^+^ cells [84]. Vα7.2^+^ cells were enriched in fibrotic, but also present in non-fibrotic areas [4, 84]. Importantly, there is a strong clinical association of PSC with inflammatory bowel disease (IBD), the latter being associated with a decreased intestinal barrier integrity, strong mucosal inflammatory responses also involving MAIT cells [38, 73], and changes in the gut microbiome [85, 86].

Patients with PBC, characterized by nonsuppurative destructive cholangitis of the small interlobular bile ducts, showed similar depletion of MAIT cells in the liver and blood [4, 87], the depletion in blood being partially reversed after ursodeoxycholic acid treatment [88]. Peripheral MAIT cells were responsive to in vitro TCR stimulation and had a more activated and apoptotic phenotype [87, 88]. Increased serum and hepatic IL-7 levels in PBC patients might be contributing to the pathogenic role of MAIT cells in this context [88]. Interestingly, the absolute number of MAIT cells in the liver was slightly increased in this study, as assessed by in situ microscopic evaluation using MR1-tetramer staining [88].

Taken together, no disease-specific pattern of MAIT cell function in autoimmune liver diseases has emerged to date. Most importantly, it is not known whether MAIT cells themselves may be involved in autoimmunity, as has been, e.g., described for mouse iNKT cells in the development of a PBC-like disease in response to bacterial triggering [89]. From available data, there is a possibility of increased frequency or absolute numbers of MAIT cells in the liver of patients with autoimmune liver diseases; this issue needs to be further investigated using more robust technologies.

### Fatty liver disease

Fatty liver disease is caused by two main etiologic factors: metabolic syndrome and excess alcohol consumption. The main histological features of steatohepatitis that include steatosis, portal and lobular inflammation, and hepatocyte ballooning cannot distinguish between these two etiologies. Nonalcoholic fatty liver disease (NAFLD), comprising both simple fat accumulation and nonalcoholic steatohepatitis (NASH), is a liver manifestation of the metabolic syndrome. The prevalence, morbidity, and mortality of this condition are increasing due to the ongoing obesity epidemic. NAFLD can lead to progressive fibrosis and development of hepatocellular carcinoma (HCC), even in a non-cirrhotic liver. There is a well-established link between the metabolic syndrome and changes in the gut-resident bacteria, and accumulating evidence suggests the involvement of the gut microbiome in the pathogenesis of various liver diseases including NAFLD [90, 91]. This renders the study of MAIT cells, which respond to compounds derived from microbial riboflavin biosynthesis intermediates, of particular interest. As obesity and type 2 diabetes are closely connected with NAFLD, the findings obtained in obese and/or diabetic patients, in which MAIT cells are less abundant in blood and produce IL-17 in adipose tissue, will possibly also reflect changes associated with fatty liver disease [92]. In obese mice, MAIT cells were reported to play an unfavorable role in the development of metabolic dysfunction, involving macrophage M polarization in adipose tissue as well as dysbiosis and gut leakiness [93]. Definitely, the relation between liver MAIT cells and alterations in gut microbiota in the context of liver diseases should receive more attention in the future.

To date, only a few studies have been conducted on MAIT cells in NAFLD. A decreased frequency of MAIT cells in the circulation of NAFLD patients was observed [5, 6, 60], with increased expression of activation markers [6]. In mice fed a methionine/choline-deficient diet, MAIT cells appeared to have a protective effect, as indicated by aggravated NASH progression in MR1-deficient mice, accompanied by increased frequency of proinflammatory macrophages [60]. The positive effect of MAIT cells reported in this study stands in contrast to the rather harmful role of MAIT cells in other mouse models of obesity and liver fibrosis [5, 93], as outlined above and in the next section. Therefore, more studies, especially focusing on the analysis of liver-derived MAIT cells, are needed to clarify their role in NAFLD.

The crosstalk between the gut microbiome and the liver, and quantitative as well as qualitative changes in the intestinal microbiome in alcohol-related liver disease (ALD) [94], make it plausible that MAIT cells are implicated in ALD development and that – inversely – ALD also influences MAIT cell function. Indeed, the loss of MAIT cells and their compromised antimicrobial potency in ALD has been linked to a leaky gut [95]. The authors proposed that the leaky gut leads to excessive contact with microbial products which render MAIT cells dysfunctional, resulting in bacterial infections; maintenance of MAIT cells might therefore be important to prevent secondary infections [95]. Other studies looking at MAIT cells in ALD also report a loss of peripheral MAIT cells [5, 96]. The highly activated and exhausted state of MAIT cells decreased with alcohol abstinence [96], but the numbers of MAIT cells did not fully normalize, demonstrating that alcohol abuse can result in significant changes to MAIT cell frequencies which may be irreversible.

In conclusion, MAIT cells likely play a relevant role in all liver diseases discussed above. In most studies, MAIT cells are described as depleted, with the remaining MAIT cells mostly in an activated state and still responsive to mainly cytokine-mediated stimulation, thus likely contributing to inflammatory processes both systemically and in the liver. Consistent with their innate-like and thus general mode of action, to date no clear liver disease-specific pattern of MAIT cell phenotype and function has been found in liver inflammation. Data on liver MAIT cells are scarce and largely descriptive and further research using cutting-edge technologies, and particularly addressing pathologies such as NAFLD and autoimmune liver diseases, in which liver MAIT cells have not yet been extensively studied, is needed to advance our understanding of the topic.

## Role of MAIT cells in the development of liver fibrosis

Liver fibrosis is the result of chronic inflammatory injury to the liver. It reflects the deposition of collagen fibers and leads to architectural changes in the liver that can ultimately lead to liver cirrhosis and associated serious complications including loss of liver function, development of portal hypertension, and HCC.

As IL-17 acts as a fibrogenic cytokine that activates HSCs/hepatic myofibroblasts (HMFs) to produce extracellular matrix proteins and consequently is crucially involved in liver fibrogenesis [97–99], a connection of MAIT cells and liver fibrosis was highly anticipated. Indeed, MAIT cells were found in vitro to stimulate proliferation and activation of HSCs/HMFs, an effect that was partially mediated by IL-17 and partially by cell-to-cell contacts [5, 6]. Additionally, the MAIT cell-secreted TNF-α led to proinflammatory cytokine secretion by HMFs [5]. Our own results on MR1-dependent MAIT cell activation by HSCs suggest that the interaction between HSCs and MAIT cells might reciprocally potentiate their pro-fibrotic properties [7]. Most importantly, the group of Sophie Lotersztajn elegantly demonstrated that the lack of MAIT cells in MR1^−/−^ mice protects against liver fibrosis in a model of carbon tetrachloride (CCl4)-induced liver injury; conversely, increased amounts of MAIT cells in MAIT-TCR transgenic mice enhanced liver fibrosis [5].

Other MAIT cell-secreted cytokines that might be involved in pro- or anti-fibrotic processes in the liver include IL-22, IL-26, and IFN-γ. The evidence for IL-22-mediated anti-fibrotic effects in the liver is quite strong; e.g., IL-22 was shown to contain liver fibrosis and promote its resolution in a mouse model of CCL4-induced liver injury and to induce HSC senescence [100]. Interestingly, IL-22 was also shown to ameliorate acute-on-chronic liver failure in mice by reprogramming impaired regeneration pathways. Whereas IL-22 secretion by liver MAIT cells was mainly observed upon TCR triggering, IL-26 secretion was induced by cytokine stimulation [30], and IL-26 was reported as pro-fibrogenic [101]. The role of IFN-γ in liver disease progression and fibrosis is more difficult to decipher as there is evidence for strong pro-inflammatory and thereby injurious action, especially in chronic cholangitis, but also increasing evidence for it being anti-fibrotic, mostly by increasing NK cell-mediated cytotoxicity towards HSCs and by inhibiting HSC proliferation (reviewed in [102]).

TNF-like ligand 1 A (TL1A), prominently expressed on myeloid cells under inflammatory conditions, is another interesting cytokine to mention in the context of fibrosis. TL1A was found to increase pro-inflammatory cytokine secretion by gut-derived MAIT cells in response to cytokine simulation [28]. As there is also evidence for it being involved in liver fibrosis in mice [103] and of its expression increased in the PBC liver (with its soluble form raised in sera of patients with chronic liver diseases) [104], it is possible that TL1A will also increase response of liver MAIT cells to inflammatory cues, thereby accelerating liver fibrosis. Clearly, additional work is needed to better understand the role of the aforementioned cytokines in liver fibrosis.

**Fig. 4:**
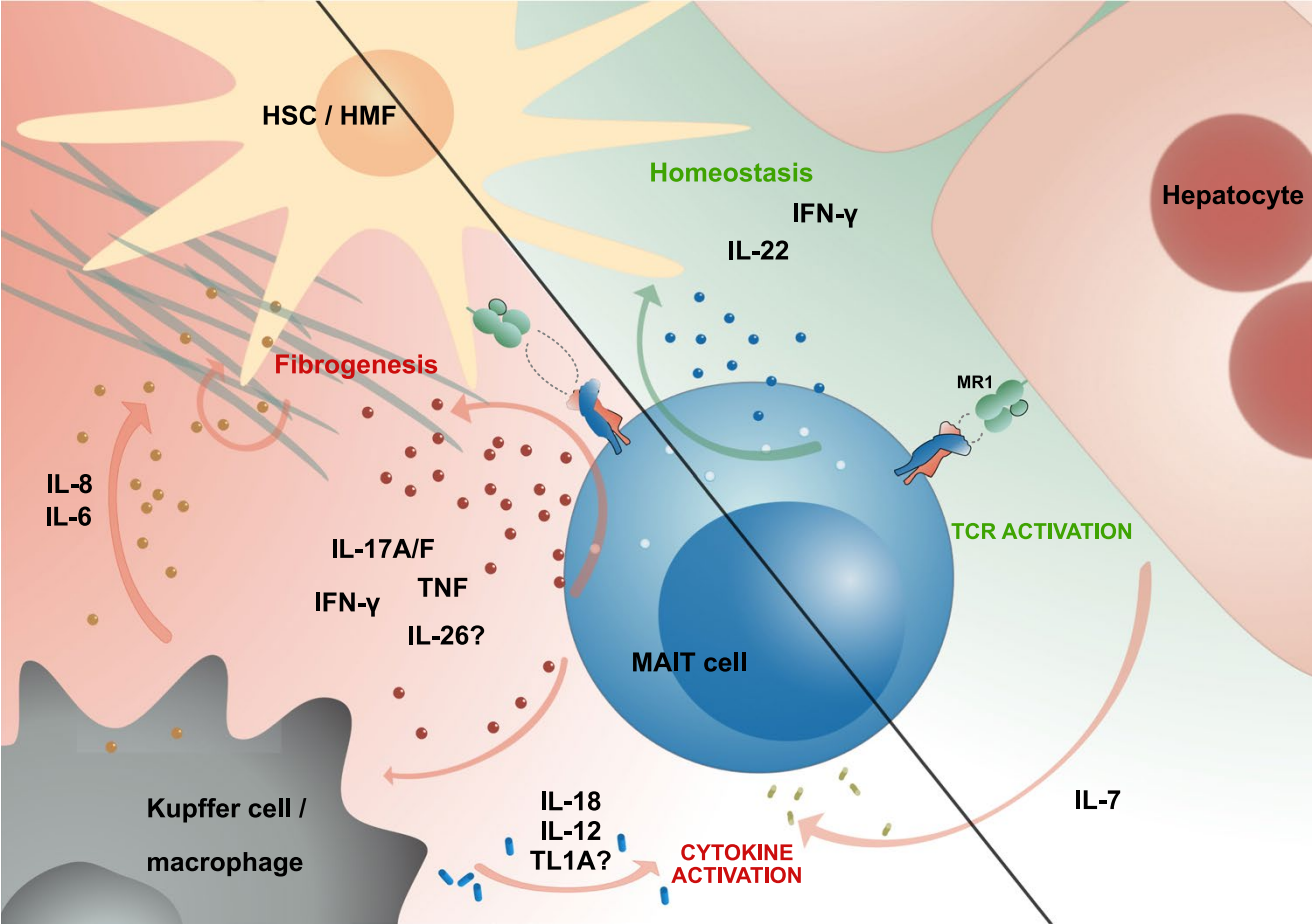
MAIT cells’ fibrogenic potential and their role in liver homeostasis and tissue repair. MAIT cells can be triggered via their TCR and/or cytokine receptors. The mode of MAIT cell activation influences their impact on fibrogenesis. TCR activation leads to the secretion of anti-fibrotic effector molecules, including IL-22 and IFN-γ; both cytokines have been shown to inhibit HSC activation. TCR-triggered MAIT cells were also shown to produce proteins involved in tissue repair. Inflammatory cytokine-dependent activation drives a strong secretion of fibrogenic cytokines. IL-7, generated by hepatocytes under inflammatory conditions, twists MAIT cells towards IL-17 secretion, while IL-18/IL-12, produced by myeloid cells, orchestrates a strong TNF/IFN-γ response. The presence of TNF and IL-17 further promotes the secretion of pro-inflammatory cytokines IL-6 and IL-8 by macrophages and hepatic stellate cells (HCSs)/hepatic myofibroblasts (HMFs). This cytokine milieu will stimulate the secretion of collagen and tissue inhibitor of metalloproteinases 1 (TIMP1) by activated myofibroblasts. In addition, evidence is accumulating towards a role of TL1A in the secretion of TNF by MAIT cells and also in liver fibrogenesis. Finally, in presence of pro-inflammatory conditions, MAIT cells can secrete IL-26, recently shown to activate HSCs. See text for references

Liver MAIT cells from patients with cirrhosis (due to either alcoholic or nonalcoholic fatty liver disease) show alterations consistent with exhausted and pro-fibrotic activity, including increased frequencies of IL-17A^+^ cells [5]. Decreased frequency of intrahepatic MAIT cells and a significant negative correlation between cell frequency and expression of PD-1 could argue for activation-induced cell death also occurring in the liver. In cirrhotic patients, Vα7.2^+^ cells were found to accumulate in fibrotic septa [5], strengthening their potential involvement in liver fibrosis; notably however, Va7.2^+^ cells were primarily located in portal fields in another study of alcohol-related cirrhosis [95]. The frequency of peripheral MAIT cells was also strongly decreased in cirrhosis, as opposed to iNKT cells and γδ cells [5]. Taken together, liver MAIT cells seem to undergo dramatic changes in the course of progressing liver fibrosis. Figure 4 highlights the fibrogenic potential of liver MAIT cells and their likely involvement in liver homeostasis and tissue repair.

Decompensated cirrhosis, clinically reflected by decreased liver function and/or increased portal pressure, is known to be associated with increased susceptibility to infections (including spontaneous bacterial peritonitis; SBP). Advanced liver disease has been linked to increased gut leakiness [105, 106], leading to increased exposure of the liver to bacteria-derived products, including endotoxins and metabolites. Indeed, a recent analysis revealed increased levels of MAIT cell stimulatory ligands in sera derived from patients with portal hypertension, a condition associated with a leaky gut barrier [7]. Interestingly, in contrast to intrahepatic MAIT cells, peritoneal MAIT cells remained abundant and highly functional in patients with decompensated cirrhosis presenting with ascites and were further enriched in SBP [107].

It is possible that already slight damage to intestinal, either epithelial or gut vascular, barrier integrity, which may not be apparent in histological or clinical studies, will lead to increased exposure of intrahepatic immune cells to gut-derived products, even before liver cirrhosis has developed, fostering intrahepatic MAIT cell activation and profibrogenic activity. Therefore, it could be speculated that modulation of the influx of bacteria-derived ligands to the circulation might represent a new approach to attenuate and prevent liver fibrosis. This might be achieved by interfering with the microbiome composition or through repressing the microbial riboflavin biosynthesis pathway, e.g., by providing riboflavin along the digestive tract. Additionally, nonabsorbable gut-selective antibiotics, e.g., rifaximin, as currently used in the management of hepatic encephalopathy (representing a severe complication of liver cirrhosis) [108], will also alter the gut microbiome composition and may potentially affect MAIT cell activation and associated liver injury.

A recent report about the ability of acetyl-6-formylpterin (acetyl-6-FP, an MR-1 antagonist) to accelerate regression of liver fibrosis in mice [109] supports the concept of using inactive MR1 ligands in the treatment of liver diseases. The effectiveness of hepatocytes and other liver cell types in presenting Ag to MAIT cells makes this approach of particular importance, both for the treatment and prevention of fibrosis [4, 7]. As MAIT cell function in humans and mice may vary substantially and intrahepatic MAIT cell frequencies also differ between these two organisms (see above), it is hard to predict the side effects of blocking MAIT cell activation in humans. Still, the prospect of modulating liver fibrosis development and/or progression by interfering with MAIT cell function is appealing and worthy of testing in future clinical studies.

## Conclusions: a model for MAIT cells in liver homeostasis and disease

We propose a model, in which MAIT cells − maintained in the healthy liver in their activated, tissue resident phenotype due to continuous low-level TCR-dependent stimulation − play a protective role in liver homeostasis. By continuously interacting with parenchymal and non-parenchymal liver cells, MAIT cells might contribute to ongoing liver regeneration and tissue repair processes, besides their obvious role in antibacterial and antiviral immune defenses. Research in this direction will likely uncover yet unrecognized interactions and mechanisms, and is being highly encouraged.

In the context of ongoing inflammation in the liver, MAIT cells will be exposed to increased inflammatory cues, including cytokines secreted by resident and infiltrating immune cells. Although being depleted in proportion and numbers due to infiltrating non-MAIT T cells, and also activation-induced cell death, the remaining MAIT cells will be activated towards a profibrogenic phenotype, likely contributing to immunopathology in the liver. Because of their granzyme B production [25], MAIT cells in this context may also contribute to cytotoxic processes.

With the advancement of single-cell technologies and the availability of novel multiplexing imaging technologies including cytometry by time of flight (CyTOF) and imaging mass cytometry (IMC), it will be possible to study complex cellular phenotyping at single-cell level as well as spatial contexts in the tissue, which should advance our current understanding of MAIT cell physiology in health and disease. Murine in vivo models can be crucial to demonstrate nonredundant roles of these cells, despite the relatively low frequencies in mice. Advances in organoid technology, including the use of HCC-derived organoids [110, 111] and cell-based as well as patient-tissue based spheroids (harboring not only hepatocytes but also endothelial cells, myofibroblasts, and Kupffer cells) [112], should allow similar mechanistic studies to be explored in human tissue, e.g., by performing co-cultures with liver-derived MAIT cells. Such studies may allow an analysis of tissue repair pathways relevant to injury and homeostasis. These approaches will also reveal novel mechanisms of immune interactions in the human liver and hopefully reveal new targets for immunotherapy and antifibrotic treatments.

## Abbreviations

5-A-RU: 5-Amino-6-D-ribitylaminouracil
5-OP-RU: 5-(2-Oxopropylideneamino)-6-D-ribitylaminouracil
Ab: Antibody
Ag: Antigen
AIH: Autoimmune hepatitis
ALD: Alcoholic liver disease
APC: Antigen presenting cell
BEC: Biliary epithelial cell
HBV: Hepatitis B virus
HCV: Hepatitis C virus
HDV: Hepatitis delta virus
HMF: Hepatic myofibroblast
HSC: Hepatic stellate cell
IBD: Inflammatory bowel disease
IFN-γ: Interferon gamma
IF: Immunofluorescence
IHC: Immunohistochemistry
IL: Interleukin
iNKT: Invariant Natural Killer T cell
LSEC: Liver sinusoidal endothelial cell
MAIT: Mucosal-associated invariant T cell
MHC: Major histocompatibility complex
MR1: MHC class I-related protein 1
NK: Natural killer
PBC: Primary biliary cholangitis
PBMCs: Peripheral blood mononuclear cells
PSC: Primary sclerosing cholangitis
TCR: T cell receptor
TNF-α: Tumor necrosis factor alpha
