# Correction to: MAIT cells in liver inflammation and fibrosis

**DOI:** 10.1007/s00281-022-00956-2

**Published:** 2022-07-25

**Authors:** Hema Mehta, Martin Joseph Lett, Paul Klenerman, Magdalena Filipowicz Sinnreich

**Affiliations:** 1 Peter Medawar Building for Pathogen Research, Nuffield Department of Medicine, University of Oxford, South Parks Rd, Oxford, OX1 3SY UK; 2 Liver Immunology, Department of Biomedicine, University Hospital Basel and University of Basel, Basel, Switzerland; 3 Translational Gastroenterology Unit, John Radcliffe Hospital, Oxford, OX3 9DU UK; 4 Department of Gastroenterology and Hepatology, Basel University Medical Clinic, Cantonal Hospital Baselland, Liestal, Switzerland

**Correction to: Semin Immunopathol**

**https://doi.org/10.1007/s00281-022-00949-1**

In Fig. 2 of this article, there was a mix-up of two author names (“Böttcher et al.” and “Hegde et al.”). The figure should have appeared as shown below.

**Fig. 2:**
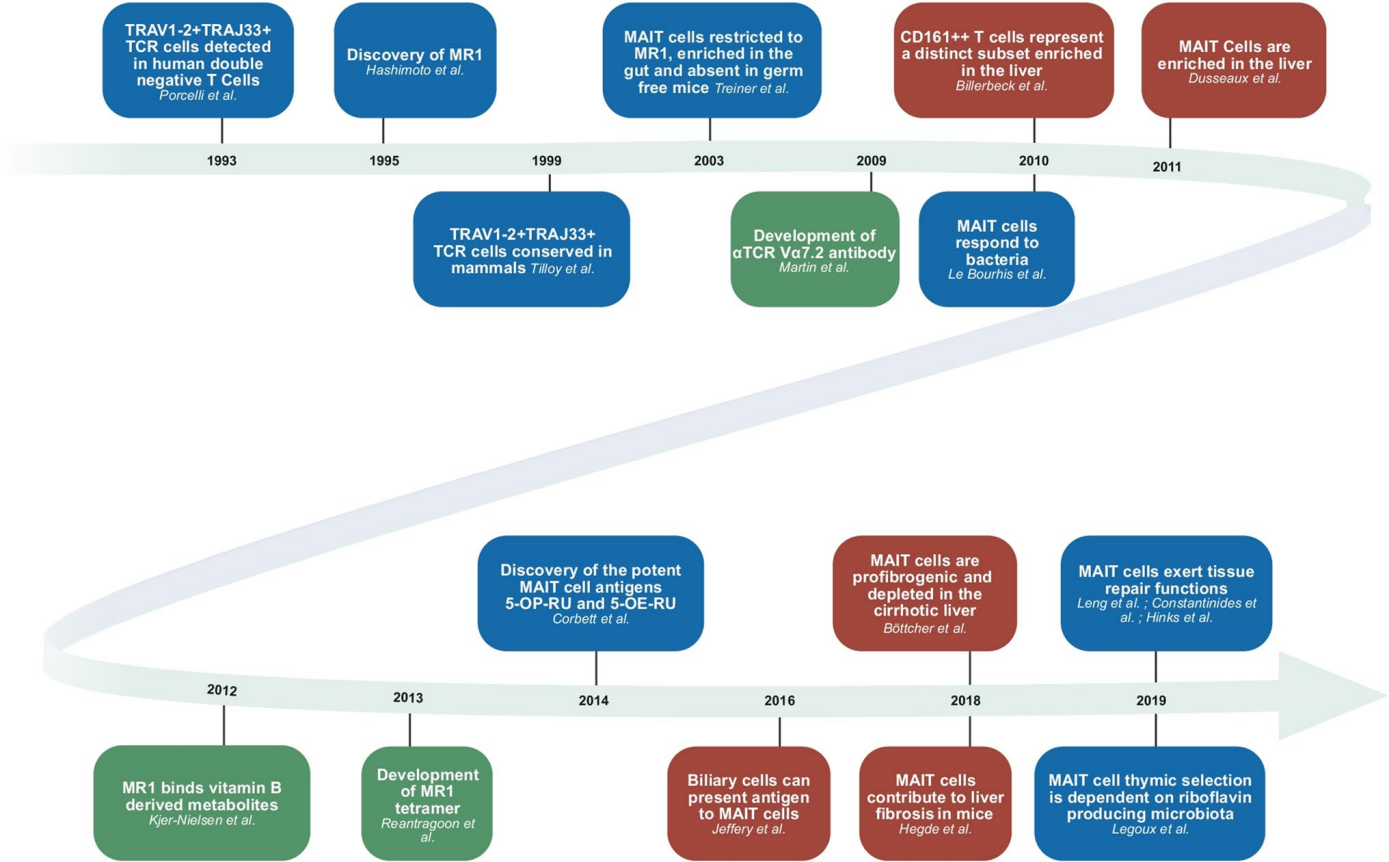
Timeline highlighting some of the most relevant findings in studies of MAIT cells in the liver. Findings in blue are crucial for the whole MAIT cell field; green boxes show crucial tool development; and studies in red concern the liver

The original article has been corrected.

